# Phylogeny of Regulators of G-Protein Signaling Genes in *Leptographium qinlingensis* and Expression Levels of Three RGSs in Response to Different Terpenoids

**DOI:** 10.3390/microorganisms10091698

**Published:** 2022-08-24

**Authors:** Tian Gan, Huanli An, Ming Tang, Hui Chen

**Affiliations:** State Key Laboratory for Conservation and Utilization of Subtropical Agro-Bioresources, College of Forestry and Landscape Architecture, South China Agricultural University, Guangzhou 510642, China

**Keywords:** *Leptographium* *qinlingensis*, regulators of G-protein signaling, terpene tolerance, host resistances, expression

## Abstract

*Leptographium qinlingensis* is a bark beetle-vectored pine pathogen in the Chinese white pine beetle (*Dendroctonus armandi*) epidemic in Northwest China. *L. qinlingensis* colonizes pines despite the trees’ massive oleoresin terpenoid defenses. Regulators of G-protein signaling (RGS) proteins modulate heterotrimeric G-protein signaling negatively and play multiple roles in the growth, asexual development, and pathogenicity of fungi. In this study, we have identified three *L. qinlingensis RGS* genes, and the phylogenetic analysis shows the highest homology with the regulators of G-protein signaling proteins sequence from *Ophiostoma piceae* and *Grosmannia clavigera*. The expression profiles of three RGSs in the mycelium of *L. qinlingensis* treated with six different terpenoids were detected, as well as their growth rates. Under six terpenoid treatments, the growth and reproduction in *L. qinlingensis* were significantly inhibited, and the growth inflection day was delayed from 8 days to 12–13 days. By analyzing the expression level of three *RGS* genes of *L. qinlingensis* with different treatments, results indicate that *LqFlbA* plays a crucial role in controlling fungal growth, and both *LqRax1* and *LqRgsA* are involved in overcoming the host chemical resistances and successful colonization.

## 1. Introduction

Phytophagous insects with fungal pathogens that attack coniferous trees have drawn a lot of attention due to the enormous amount of damage they cause. The damage caused by these fungal pathogens has important implications for climate change through the obvious impact of tree mortality on forest carbon dynamics [1]. The ascomycete *Leptographium qinlingensis* (Lq) is a fungal pathogen of Chinese white pine trees (*Pinus armandii*) and is vectored by the Chinese white pine beetle (*Dendroctonus armandi*, CWPB) [2]. CWPB and Lq form an interactive biological complex that has caused a rapid, large-scale decline in the Chinese white pine (*P. armandii*) in the Qinling and Bashan Mountains of China [3,4,5]. *L. qinlingensis* can stain the sapwood blue when the CWPB-Lq complex is inoculated manually into healthy host trees; such discoloration reduces the commercial value of lumber [6,7,8]. Like all conifers, the pine hosts of the CWPB-Lq complex have complex oleoresin-based chemical defences that protect these trees against most potential pests and pathogens [9,10]. The highly specialized CWPB-Lq complex, which colonizes the monoterpene-rich environment of pine phloem and sapwood, requires mechanisms to overcome the host defence chemicals [11,12].

The heterotrimeric G-protein (G-protein) signaling pathway is one of the most important signaling pathways that transmits external signals into the inside of the cell [13]. G-proteins are composed of α (Gα), β (Gβ), and γ (Gγ) subunits. The β and γ subunits are tightly associated and can be regarded as one functional unit [14,15]. In the presence of a ligand, G-protein-coupled receptors (GPCRs) activate the G-proteins through the exchange of GDP for GTP on the Gα subunit, resulting in the dissociation of the Gα and Gβγ complex [16,17]. Both Gα and Gβγ subunits signal to various cellular pathways [14]. Upon GTP hydrolysis to GDP by the intrinsic GTPase activity of the Gα subunit, the G-protein subunits reassociate, and signaling is terminated [18,19]. In addition, regulators of G-protein signaling (RGS) proteins are a group of proteins containing a conserved RGS domain of about 120 amino acids, which specifically interacts with the GTP-bound Gα subunit, accelerates its intrinsic GTPase activity, and thus negatively regulates G-protein signaling [19,20].

In filamentous fungi, four RGS proteins (*Sst2*, *Rgs2*, *Rax1*, and *Mdm1*) were first found in *Saccharomyces cerevisiae* [21,22]. In the genome of the model filamentous fungus *Aspergillus nidulans*, five genes (*FlbA*, *RgsA*, *RgsB*, *RgsC*, and *GprK*) that encode RGS proteins were identified. The functions of these *A. nidulans RGS* genes in controlling hyphal proliferation, asexual spore formation, sexual fruiting, and the mycotoxin sterigmatocystin production have been discovered [23,24,25]. *Aspergillus flavus* contains six RGS domain-containing proteins (*RgsA*, *RgsB*, *RgsC*, *RgsD*, *RgsE*, and *FlbA*) and has been established as having important roles in pathogenicity [26]. Likewise, the functions of six RGSs (*FlbA*, *GprK*, *RgsA*, *Rax1*, *RgsC*, and *RgsD*) from *Aspergillus fumigatus* in the regulation of fungal growth, asexual development, germination, stress tolerance, and virulence have been identified [17,27,28,29,30]. These studies demonstrate that RGSs of filamentous fungi are involved in multiple important biological processes. However, the role of RGSs in overcoming host resistance and successful colonization during phytopathogenic fungi invasion of host trees remains unclear.

In this report, we identify three *RGS* genes from *L. qinlingensis* and compare their homology with other fungal RGSs. Based on the results of this experiment, three concentrations (5%, 10%, and 20%) of (±)-α-pinene, (−)-β-pinene, (+)-3-carene, (+)-limonene, turpentine, and mix-monoterpene are used to amend artificial media to determine their effects on the growth and reproduction (measured as colony area) of *L. qinlingensis*. In addition, the expression of three *LqRGS* genes under different terpenoid treatments confirms the role of *RGS* genes in *L. qinlingensis* overcoming the resistance of host trees.

## 2. Materials and Methods

### 2.1. Strain

*Leptographium qinlingensis* (NCBI Taxonomy ID: 717,526) was isolated from *P. armandii* sapwood phloem that had been attacked by the *D. armandi* in the Qinling Mountains and deposited at the College of Forestry and Landscape Architecture, South China Agricultural University (Guangzhou, China).

### 2.2. Fungal Media and Growth Condition

*L. qinlingensis* was cultured on potato dextrose agar (PDA) media in the dark for 7 days at 28 °C for induction. A disk (1 cm diameter) was cut using a cork borer from the actively growing margin of the source of fungus and transferred to the center of MEA medium containing 1% Oxoid malt extract agar and 1.5% technical agar overlaid with cellophane, and the pH was adjusted to 5.5 for subsequent experiments.

### 2.3. Terpenoid Treatments

Experimental group: Monoterpenes (±)-α-pinene((+)-α-pinene:(−)-α-pinene = 1:1), (−)-β-pinene, (+)-3-carene, (+)-limonene, turpentine, mix-monoterpene ((+)-limonene: (+)-3-carene: (±)-α-pinene: (−)-β-pinene = 5:3:1:1) were diluted with DMSO to three concentrations of 5%, 10% and 20% [31,32,33]. The main steps of terpenoid treatment are shown in Supplementary Figure S1. According to the purity of the terpenoid reagent, the volume (V1) of DMSO to be added to each 100 mL stock solution when preparing a 20% concentration terpenoid dilution was calculated (Table 1). Add 100 mL of terpenoid stock solution and V1 volume of DMSO to the burette, and after mixing, it is a 20% concentration of terpenoid dilution (solution A). Take 100 mL of solution A into a new burette, add 100 mL of DMSO, and mix to obtain a 10% concentration of terpenoid dilution (solution B). Take 100 mL of solution B into a new burette, add 100 mL of DMSO, and mix to obtain a 5% terpenoid dilution (solution C). Pipette 200 mL of each concentration test chemical onto the centre of each MEA medium overlaid with cellophane and gently swirl over the agar surface before inoculation.

Control group: 200 mL DMSO was added to the MEA medium overlaid with cellophane.

A 1 cm diameter *L. qinlingensis* mycelial plug was inserted into the center of the above medium, incubated at 28 °C in the dark, and the growth (colony area measured by its diameter in cm) was measured every 3 days in four directions and averaged until the strain brought the fungus to the edge of the plate [32,34]. For the eight different media, the growth condition was obtained by calculating the area of the colony. Each treatment was repeated five times.

### 2.4. RNA Isolation and cDNA Synthesis

Total RNA was isolated from *L. qinlingensis* by the UNIQ-10 Column Trizol Total RNA Isolation Kit (Sangon Biotech, Shanghai, China) following the manufacturer’s protocol. Its integrity was checked on 1% agarose gels and quantified using NANO DROP 2000 spectrophotometry (Thermo Scientific, Pittsburgh, PA, USA). The purity was calculated by the mean of relation A260/A280 ratio (μg/mL = A260 × dilution factor × 40). The synthesized cDNA obtained from the sample was used as the template using the HisScript^®^III 1st Strand cDNA Synthesis Kit (+gDNA wiper) (Vazyme Biotech, Nanjing, China).

### 2.5. Gene Amplification and Cloning

The cDNA synthesized from the sample was used as a template for the PCR reaction. Degenerate primers (Table 2) were designed in Primer Premier 5.0, based on the regulator of G-protein signaling (RGS) protein sequences of *Grosmannia clavigera* and *Ophiostoma piceae* from NCBI (http://www.ncbi.nlm.nih.gov/, accessed on 18 July 2022). PCR amplifications were performed in a C1000 Touch Thermal Cycler (Bio-Rad, Hercules, CA, USA), and the cDNA amplification was performed in a 50 μL reaction volume: 5 μL cDNA, 10 μM each primer, 25 μL Green Taq Mix (Vazyme Biotech, Nanjing, China), with ddH_2_O added to 50 μL. The reaction conditions were as follows: 95 °C for 5 min, 30 cycles of 95 °C for 30 s, TM of each pair of primers for 30 s and 72 °C for 1 min, with a final extension for 10 min at 72 °C. The PCR products were visualized on 1% agarose gels stained with Ultra GelRed (10,000×) (Vazyme Biotech, Nanjing, China) and compared with a DL2000 Plus DNA Marker (Vazyme Biotech, Nanjing, China).

Single-stranded 5’ and 3’ RACE-ready cDNA was synthesized from RNA using a SMARTer™ RACE cDNA Amplification Kit (Clontech Laboratories Inc., Mountain, CA, USA) according to the manufacturer’s protocol. Partial sequences were used in the primer design (Table 2), and the PCR was performed as described in the SMARTer™ RACE cDNA Amplification Kit (Clontech Laboratories Inc., Mountain, CA, USA). The amplicons were purified, cloned, and sequenced. Sequences were manually edited with DNAMAN 6.0 software (Lynnon BioSoft, Vaudreuil, Quebec, Canada) to obtain inserts sequences, which were then BLASTed against the NCBI database.

### 2.6. Analysis of Full-Length cDNA Sequences

Full-length cDNA sequences were assembled in DNAMAN 6.0, using sequence fragments and RACE results. To avoid chimera sequences, specific primers (Table 2) from initiation to terminator codon were designed based on complete sequences. Open reading frames (ORFs) of full-length cDNA were obtained via ORF Finder (https://www.ncbi.nlm.nih.gov/orffinder/, accessed on 18 July 2022), and cDNA was then translated to amino acid sequences using the ExPASy Translate Tool (http://www.expasy.org/tools/dna.html, accessed on 18 July 2022), and colored in DNAMAN 6.0. Molecular mass (kDa) and isoelectric points were determined in the ProtParam tool. RGSs of *L. qinlingensis* were checked for likely subcellular localization using Target P 2.0 software (http://www.cbs.dtu.dk/services/TargetP/, accessed on 18 July 2022) with the default parameters. RGSs of *L. qinlingensis* homologs were identified with the NCBI-BlastP network server (https://blast.ncbi.nlm.nih.gov/Blast.cgi, accessed on 18 July 2022). Amino acid identity was analyzed through the construction of a homology tree in DNAMAN6.0. A neighbor-joining phylogenetic tree was built in MEGA-X, employing ClustalW with default parameters, p-distance model, pairwise gap deletion, and 1000 bootstrap replicates. The putative N-terminal signal peptide was predicted in SignalP 5.0 Server (https://services.healthtech.dtu.dk/service.php?SignalP-5.0, accessed on 18 July 2022).

### 2.7. Effects of Terpenoids on Expression Levels of three L. qinlingensis RGSs (Real Time-qPCR)

In the Ultra-clean workbench, the mycelium cultured for 15 days on the solid medium was gently scraped with tweezers into the RNase-free microfuge tubes (1.5 mL) and stored at −80 °C until use. RNA isolation and cDNA synthesis followed previous descriptions (“RNA Isolation and cDNA Synthesis”). Five repetitions per treatment were prepared.

The CFX96TM Real-Time PCR Detection System (Bio-Rad, Hercules, CA, USA) was used for qRT-PCR, with *L. qinlingensis EF1* (accession number: AHZ56579.1) as the reference gene. Specific qRT-PCR primers were designed in PRIMER 3Plus (https://www.primer3plus.com/, accessed on 18 July 2022), based on nucleotide sequences (Table 2), and their amplification efficiencies were calculated using relative standard curves with a five-fold cDNA dilution series; the efficiency values for the primers were 100 ± 5%. The sizes of the amplicons were 128 bp (*EF1*), 135 bp (*FlbA*), 127 bp (*Rax1*), and 136 bp (*RgsA*). Amplicons were confirmed as the correct size after the qRT-PCR assay via gel electrophoresis and were then sequenced by a biotechnology company (TSINGKE Biotechnology, Beijing, China) to make sure the correct amplification products were obtained.

The reaction mixture (20 µL) contained 10 µL of ChamQ Universal SYBR qPCR Master Mix (Vazyme Biotech, Nanjing, China), 2 µL of cDNA (diluted 4 times), 0.4 µL of each primer, and 7.2 µL of nuclease-free water. Template-free negative controls were included in every reaction. Thermocycling conditions were as follows: 95 °C for 2 min, followed by 39 cycles of 95 °C for 10 s, and 60 °C for 30 s, melting curve analysis at 95 °C for 5 s, 65 °C for 5 s. At the end of each reaction, a melting curve analysis was performed to detect single gene-specific peaks and check for primer dimers. Three biological and three technical replicates were included to ensure reproducibility.

### 2.8. Statistical Analysis

Relative expression values of *L. qinlingensis* RGSs were determined using the Ct (ΔΔCt) method (Livak and Schmittgen 2008) and analyzed with Excel 2019 (Microsoft Office). To evaluate significant differences in the expression for *L. qinlingensis* RGSs, 2^−ΔΔCt^ values transformed at log_2_ were subjected to a one-way analysis of variance (ANOVA) and Tukey’s honest significant difference test (HSD) to determine whether the gene expression differed among the treatments. The 2^−ΔΔCt^ values and standard error (SE) were transformed at log_2_ to generate graphs. All of the statistical analyses were performed with SPSS 22.0 (IBM SPSS Statistics, Chicago, IL, USA) and plotted with OriginPro 2021 software (OriginLab, Northampton, MA, USA).

## 3. Results

### 3.1. Identification of L. qinlingensis RGS Genes

In *L. qinlingensis*, three regulators of G-protein signaling (RGS) genes were identified, all of which possess the conserved RGS domain and were termed *LqFlbA*, *LqRax1*, and *LqRgsA*. The full-length sequence of RGSs in *L. qinlingensis* showed a relatively high amino acid identity to the sequences predicted from the genomes of the bark beetle-associated fungi *O. piceae* UAMH 11346 and *G. clavigera* kw1407, respectively, *LqFlbA* (87.98% and 77.18%), *LqRax1* (92.35% and 83.74%), *LqRgsA* (79.38% and 65.87%). RGSs in *L. qinlingensis* also shared a strong identity with other RGSs genes from the genus *Sporothrix* (*S. brasiliensis*, *S. insectorum*, and *S. schenckii*) (Table 3).

**Figure 1:**
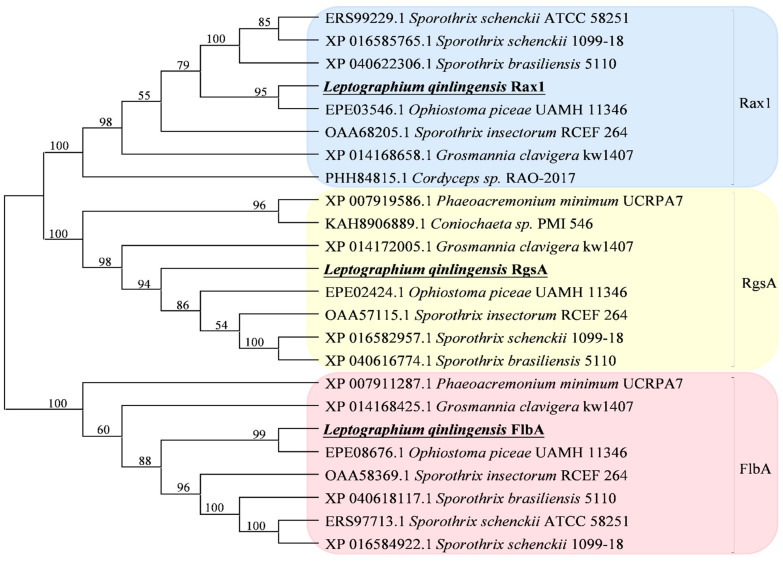
Phylogenetic tree of three regulators of G-protein signaling (RGS) from *L. qinlingensis*. The sequences from *L. qinlingensis* are marked in black. The phylogenetic tree was constructed with MEGA-X using the neighbor-joining method. Values indicated at the nodes are bootstrap values based on 1000 replicates.

Phylogenetic analysis revealed that these three *L. qinlingensis* RGSs amino acid sequences had the highest identity to *O. piceae* UAMH 11346 according to a neighbor-joining method analysis of the putative full-length amino acid sequences (Figure 1).

### 3.2. Physicochemical Properties and Bioinformation Analysis

The full-length open reading frames (ORFs) of RGSs in *L. qinlingensis* were 2349 bp (*LqFlbA*), 1101 bp (*LqRax1*), and 1296 bp (*LqRgsA*) encoding 782 (*LqFlbA*), 366 (*LqRax1*), and 431 (*LqRgsA*) amino acids. The predicted molecular masses were 85.09 kDa (*LqFlbA*), 41.23 (*LqRax1*), and 48.46 kDa (*LqRgsA*). The lowest isoelectric point of RGSs was 5.91 (*LqRax1*), and the isoelectric point of *LqFlbA* and *LqRgsA* were 9.04 and 9.60, respectively. The predicted subcellular location of *LqFlbA* and *LqRax1* by the Target P1.1 program suggests a cytoplasmic location, but *LqRgsA* suggests a nuclear location (Table 4).

**Figure 2:**
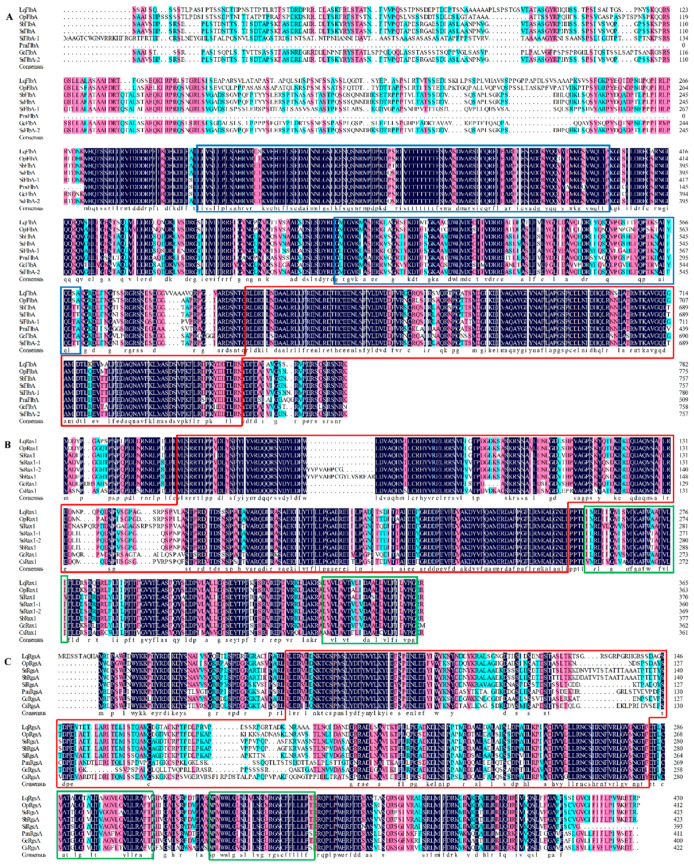
Structure of RGSs in *L. qinlingensis*. (**A**) Alignment of *LqFlbA* sequences and the consensus sequences in other species, including: *O. piceae* UAMH 11346 (*OpFlbA*, EPE08676.1), *S. brasiliensis* 5100 (*SbFlbA*, XP_040618117.1), *S. schenckii* ATCC 58251 (*SsFlbA-1*, ERS97713.1), *S. schenckii* 1099-18 (*SsFlbA-2*, XP_016584922.1), *S. insectorum* RCEF 264 (*SiFlbA*, OAA58369.1), *P. minimum* UCRPA7 (*PmFlbA*, XP_007911287.1), and *G. clavigera* kw1407 (*GcFlbA*, XP_014168425.1), both of which contain two copies of the DEP domain and a C-terminal RGS domain; (**B**) Alignment of *LqRax1* sequences and the consensus sequences in other species, including: *O. piceae* UAMH 11346 (*OpRax*1, EPE03546.1), *S. insectorum* RCEF 264 (*SiRax1*, OAA68205.1), *S. schenckii* ATCC 58251 (*SsRax1-1*, ERS99229.1), *S. schenckii* 1099-18 (*SsRax1-2*, XP_016585765.1), *S. brasiliensis* 5100 (*SbRax1*, XP_040622306.1), *G. clavigera* kw1407 (*GcRax1*, XP_014168658.1), and *C.* sp. RAO-2017 (*CsRax1*, PHH84815.1), both of which contain an N-terminal RGS domain and three DEP domains transmembrane domains; (**C**) Alignment of *LqRgsA* sequences and the consensus sequences in other species, including: *O. piceae* UAMH 11346 (*OpRgsA*, EPE03546.1), *S. insectorum* RCEF 264 (*SiRgsA*, OAA68205.1), *S. schenckii* ATCC 58251 (*SsRgsA-1*, ERS99229.1), *S. schenckii* 1099-18 (*SsRgsA-2*, XP_016585765.1), *S. brasiliensis* 5100 (*SbRgsA*, XP_040622306.1), *G. clavigera* kw1407 (*GcRgsA*, XP_014168658.1). and *C.* sp. PMI_546 (*CsRgsA*, KAH8906889.1), both of which contain an N-terminal RGS domain and three DEP domains transmembrane domains and are similar to that of Rax1. Identical amino acid residues in all proteins are shown in black; pink parts indicate more than 75% identical amino acids, and blue parts indicate more than 50% identical amino acids. Rad frames: RGS domains; Blue frames: DEP domains; Green frames: transmembrane region.

The deduced amino acid sequences and domain structures of RGSs in *L. qinlingensis* were analyzed (Figure 2 and  Figure S2). *LqFlbA* contains a C-terminal RGS domain and two DEP domains at the N-terminus (Figure 2A), which is the same as the *Sst2* in *S. cerevisiae* [35]. *LqRax1* contains an N-terminal RGS domain and three DEP domains transmembrane domains at the C-terminus (Figure 2B), which is the same as the *Rax1* in *S. cerevisiae* [36]. The structure of *LqRgsA* is similar to that of *LqRax1* (Figure 2C).

### 3.3. Effect of Terpenoids on L. qinlingensis Growth and Reproduction

**Figure 3:**
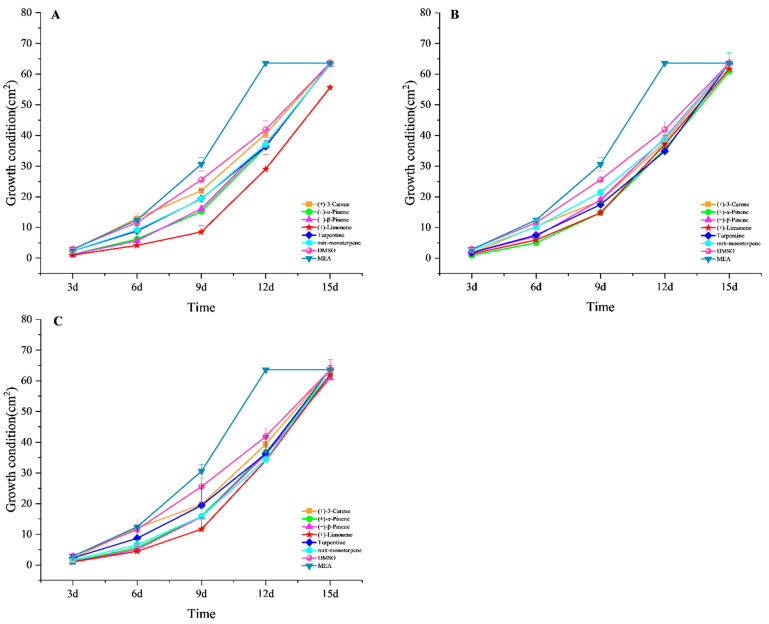
Growth rate of *L. qinlingensis* in different terpenoid treatments. The growth condition was obtained by calculating the area of the colony. The results represent the mean ± SE of five independent experiments. (**A**) 5% concentration treatments; (**B**) 10% concentration treatments; (**C**) 20% concentration treatments.

Compared with the control group (DMSO), the terpenoids showed different degrees of *L. qinlingensis* growth inhibition (Figure 3). At 5% concentration, (+)-limonene, (±)-α-pinene, and (−)-β-pinene had stronger inhibitory effects on mycelial growth than mix-monoterpene, (+)-3-carene, and turpentine (Figure 3A). At 10% concentration, (±)-α-pinene, (+)-limonene, and turpentine had stronger inhibitory effects on mycelial growth than (−)-β-pinene, (+)-3-carene, and mix-monoterpene (Figure 3B). At 20% concentration, (+)-limonene, mix-monoterpene, and (−)-β-pinene had stronger inhibitory effects on mycelial growth than (±)-α-pinene, (+)-3-carene, and turpentine (Figure 3C). In addition, the addition of DMSO alone also inhibited the growth in *L. qinlingensis* when compared to the MEA medium (Figure 3).

The logistic curve fits the growth curve of *L. qinlingensis* in different terpenoid treatments (R^2^ > 0.97). According to the logistic curve fitting of the growth curve, the growth inflection day of *L. qinlingensis* growth on MEA media occurred after approximately 9 days and the growth inflection day on different terpenoid treatments occurred after approximately 12–13 days (Table 5). However, the growth inflection day occurred after over 15 days for the medium with 5% concentration (+)-limonene treatment (Table 5).

### 3.4. Effect of Terpenoids on Expression Level of LqRGS

To determine whether the three *L. qinlingensis* RGSs are involved in overcoming host chemoresistance, we analyzed the expression profiles of RGSs from mycelia grown on MEA medium treated with six terpenoids ((+)-3-carene, (±)-α-pinene, (−)-β-pinene, (+)-limonene, mix-monoterpene, and turpentine) at three concentrations. The transcription levels in most RGSs were significantly changed after exposure to the terpenoids (Table 6).

**Figure 4:**
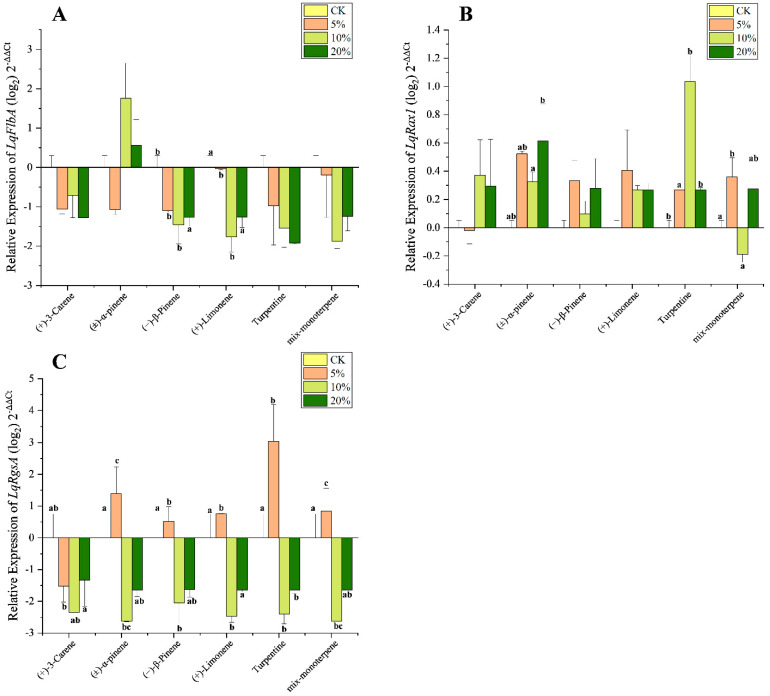
Quantitative expression of the three RGS genes (mean ± SE) in *L. qinlingensis* following treatment with different terpenoids. (**A**) Relative expression of *LqFlbA*; (**B**) Relative expression of *LqRax1*; (**C**) Relative expression of *LqRgsA*. RGSs expressions were normalized with *EF1* gene. The 2^−ΔΔCt^ and SE values were transformed at log_2_ for plotting. Different letters indicate significant differences at *p* < 0.05 (Tukey test, no letter means no significant difference) among different concentrations of the same stimulus. Mycelial growth on MEA medium with 200 mL DMSO was set as the control group (CK). Based on the value of CK (*X*-axis), the expression levels of other treatments are higher than CK as a positive value (expression up-regulation), and vice versa for a negative value (expression down-regulation).

For *LqFlbA*, significant downregulation was found only after treatment with (−)-β-pinene and (+)-limonene at the 5% and 10% concentrations (Figure 4A). The transcription level in *LqRax1* was significantly overexpressed after treatment with (±)-α-pinene and turpentine but downregulated after treatment with mix-monoterpene at 10% (Figure 4B). The expression in *LqRgsA* was significantly downregulated after treatment with (+)-3-carene at all concentrations; however, treatment with the other five terpenoids caused overexpression at 5% but downregulation at 10% and 20% for *LqRgsA* (Figure 4C).

## 4. Discussion

As the primary upstream components of the G-protein signaling pathway, regulators of G-protein signaling (RGSs) are the key negative regulators of the G-proteins to control the activities of GTPase in Gα subunits [17,26,37]. It has been known that RGSs are highly conserved in most filamentous fungi and play diverse roles in the growth, reproduction, and pathogenicity of fungi [38,39]. In this study, three RGS genes in the bark beetle-associated fungi *L. qinlingensis* were identified, all of which possess the conserved RGS domain, and were termed *FlbA*, *Rax1*, and *RgsA*, respectively.

The *FlbA* gene from *L. qinlingensis*, homologous to *Sst2* from *S. cerevisiae* and *FlbA* from the model filamentous fungus *A. nidulans*, has the same domains including one RGS and two DEP [24,40]. The DEP domain is responsible for specific GPCRs recognition and targets RGS proteins to the Golgi and plasma membranes [35,41]. In this study, both mycelial growth and *FlbA* expression of *L. qinlingensis* were inhibited after exposure to the terpenoids (Figure 4A), which is consistent with studies on the positive regulation of asexual development by *FlbA* in other fungi such as *A. nidulans* and *A. flavus* [26,42].

As a fungal-specific RGS protein, *Rax1* has no mammalian counterparts [24]. The *Rax1* from *L. qinlingensis*—which contains one RGS domain and three putative transmembrane domains at the C-terminus—is similar to the *Rax1* protein in *S. cerevisiae* [43], which plays a key role in yeast bipolar budding reproduction [36,44,45]. In the opportunistic human pathogenic fungus *A. fumigatus*, *Rax1* has been reported to play a positive controlling role in the growth, development, and oxidative stress response [46], as well as helping to regulate the normal growth in fungi under ER stress conditions [47]. The transcription level in *LqRax1* was significantly overexpressed after treatment with (±)-α-pinene and turpentine but was downregulated after treatment with mix-monoterpene at 10% (Figure 4B). The differences in the functional expression in *Rax1* between plant pathogenic fungi and mammalian pathogenic fungi require further comparative study.

Except for the isoelectric point, the properties and functional domains of *LqRgsA* are similar to that of *LqRax1*. The bark beetle-associated fungi *O. piceae* UAMH 11346 and *G. clavigera* kw1407 also contain two *RGS* genes of similar size and structure [33,48], which belong to different groups in the phylogenetic tree (Figure 1). *LqRgsA* was expressed more significantly than *LqRax1* in response to terpenoid stress. The expression in *LqRgsA* was upregulated at low concentrations (5%) and downregulated at high concentrations (10% and 20%) in almost all terpenoid treatments except that (+)-3-carene caused downregulation at all concentrations (Figure 4C). *LqRgsA* might contribute to the fungus’ ability to overcome host defense chemicals and survive in an unfavorable environment at the beginning of the invasion of the insect-fungal complex in the host trees [8].

The beetle-fungi complex requires mechanisms to overcome host defences when colonizing healthy host trees [8,48]. Beetle-symbiotic fungi can not only help metabolize phenolics and terpenoids [49,50], but also provide nutritional support for bark beetles [34,51]. In this study, the growth rate in *L. qinlingensis* was slowed down after exposure to terpenoids, and the logistic curve showed that its growth inflection point was delayed by 4–5 days, which is consistent with the results of previous studies [31,32]. 

Overall, our results demonstrate that there are three *RGS* genes in *L. qinlingensis*, containing conserved RGS domains. The physicochemical properties and phylogenetic analysis showed that the three RGS genes in *L. qinlingensis* had high homology with the *RGS* genes in the beetle-symbiotic fungi *O. piceae* and *G. clavigera*. To explore the role of *RGS* genes in beetle-fungal complexes against host resistance, we diluted six terpenoids into three concentrations to treat *L. qinlingensis* separately. By monitoring the growth rate in hyphae exposed to host-volatile terpenoids and measuring the expression in three *LqRGS* genes, it was shown that *LqRGS* genes play an important role in both growth and overcoming host resistance.

## Figures and Tables

**Table 1:** Description of the main reagents used in terpenoid treatments.

Reagents	Reagent Source	Purity	V1 (DMSO) *
(+)-α-pinene/(−)-α-pinene	Shanghai Aladdin Bio-Technology Co., Ltd. (Shanghai, China)	98%	390 mL
(+)-3-carene	Shanghai Aladdin Bio-Technology Co., Ltd. (Shanghai, China)	90%	350 mL
(−)-β-pinene	Shanghai Aladdin Bio-Technology Co., Ltd. (Shanghai, China)	99%	395 mL
(+)-limonene	Shanghai Aladdin Bio-Technology Co., Ltd. (Shanghai, China)	98%	390 mL
turpentine	Shanghai Aladdin Bio-Technology Co., Ltd. (Shanghai, China)	AR	400 mL

* V1 represents the volume of DMSO that needs to be added to 100 mL of terpenoid reagent stock solution when preparing a 20% terpenoid dilution for the first time.

**Table 2:** Primer sequences used in the research.

Gene	Primer Direction 5′→3′		Purpose
	Forward	Reverse	
*FlbA*	ACTTGCGCGAGACCCA	AATTTCGGCACCGAGTCGCT	cDNA
	GCCAGGACGGTGCTATGAT	CGCGGATCCTCCACTAGTGATTTCACTATAGG	3′RACE
	CTAATACGACTCACTATAGGGCAAGCAGTGGTATCAACGCAGAGT	AAAGACAGGTTCTCCTCGCAG	5′RACE
	ATGTCCGCCATCTCTCAGTCCTCTT	TTACTTGCGGTTCGACCGGCTCTGG	Full-length
	GCTATGATCGACACCCTGAAG	GTAGTTTCTCAGCGTATGGTCG	qPCR
*Rax1*	CTGGCTCGATGTTGCCCAGCACATG	ACTGGAACGAGGCCAGAAAGTACAC	cDNA
	CTGCCTTTGTGCTCATCTT	CGCGGATCCTCCACTAGTGATTTCACTATAGG	3′RACE
	CTAATACGACTCACTATAGGGCAAGCAGTGGTATCAACGCAGAGT	GCTGAGGTCGCCCAGGTTC	5′RACE
	ATGGACGACTACCCCGGCG	CTACAGCCGGCGGC	Full-length
	CACCGGTCGATCTGTACTCG	CGCAGTTCACGCACATAGTG	qPCR
*RgsA*	ACCCCGCCCACCTCAAGCCC	TGGGCGTCCTTGACGCGCAGCTT	cDNA
	GCTTCTTCTTACTGCTGTTCA	CGCGGATCCTCCACTAGTGATTTCACTATAGG	3′RACE
	CTAATACGACTCACTATAGGGCAAGCAGTGGTATCAACGCAGAGT	AAAGGTGCCATTGCCGACG	5′RACE
	ATGCGAGAATCATCAACAG	TTAAAGAGGCCGCGTCTC	Full-length
	ACAAGAAGCCAGAGTACCGC	TTGTTCTCGAGGACACGCTC	qPCR
*EF1*	CCGCTGGTACGGGTGAGTT	CTTGGTGGTGTCCATCTTGTT	qPCR

**Table 3:** Amino acid identity of regulators of G-protein signaling genes isolated from *L. qinlingensis* with related sequences in other fungi species.

Gene	BLAST Matches in Genbank			Identity in the Full Length *
	Species	Gene	Accession No.	Blastp (%)
*LqFlbA*	*Ophiostoma piceae* UAMH 11346	*FlbA*	EPE08676.1	87.98
	*Sporothrix brasiliensis* 5110	*FlbA*	XP_040618117.1	83.63
	*Sporothrix schenckii* ATCC 58251	*FlbA*	ERS97713.1	83.12
	*Sporothrix schenckii* 1099-18	*FlbA*	XP_016584922.1	82.86
	*Sporothrix insectorum* RCEF 264	*FlbA*	OAA58369.1	81.89
	*Phaeoacremonium minimum* UCRPA7	*FlbA*	XP_007911287.1	79.64
	*Grosmannia clavigera* kw1407	*FlbA*	XP_014168425.1	77.18
*LqRax1*	*Ophiostoma piceae* UAMH 11346	*Rax1*	EPE03546.1	92.35
	*Sporothrix insectorum* RCEF 264	*Rax1*	OAA68205.1	86.02
	*Sporothrix schenckii* ATCC 58251	*Rax1*	ERS99229.1	88.25
	*Sporothrix schenckii* 1099-18	*Rax1*	XP_016585765.1	86.13
	*Sporothrix brasiliensis* 5110	*Rax1*	XP_040622306.1	84.6
	*Grosmannia clavigera* kw1407	*Rax1*	XP_014168658.1	83.74
	*Cordyceps* sp. RAO-2017	*Rax1*	PHH84815.1	79.78
*LqRgsA*	*Ophiostoma piceae* UAMH 11346	*Rgs*	EPE02424.1	79.38
	*Sporothrix brasiliensis* 5110	*Rgs*	XP_040616774.1	75.41
	*Sporothrix schenckii* 1099-18	*Rgs*	XP_016582957.1	74.70
	*Sporothrix insectorum* RCEF 264	*Rgs*	OAA57115.1	72.84
	*Grosmannia clavigera* kw1407	*Rgs*	XP_014172005.1	65.87
	*Phaeoacremonium minimum* UCRPA7	*Rgs*	XP_007919586.1	63.25
	*Coniochaeta* sp. PMI_546	*Rgs*	KAH8906889.1	61.31

* As predicted by BLAST (www.ncbi.nlm.nih.gov, accessed on 18 July 2022).

**Table 4:** Physicochemical properties and cellular localization of RGSs of *L. qinlingensis*.

Gene Name	Full Length (bp) *	ORF Size (aa/bp) *	Mw (kDa) *	I.P. *	Signal Peptide Prediction **
*LqFlbA*	2699	782/2349	85.09	9.04	SP 0 mTP 0 other 1
*LqRax1*	1884	366/1101	41.23	5.91	SP 0 mTP 0 other 1
*LqRgsA*	1894	431/1296	48.46	9.60	SP 0.0001 mTP 0.0001 other 0.9998

* As predicted by the ProtParamprogram. ** As predicted by TargetP 2.0 program. I.P.: isoelectric point; Mw: molecular weight; ORF: open reading frame; SP: secretory pathway signal peptide; mTP: mitochondrial targeting peptide.

**Table 5:** Growth curve of *L. qinlingensis* in different terpenoid treatments after logistic curve fitting.

Media		K	a	b	R2	Inflection Day
DMSO		91.167	45.003	4.625	0.997	12.35
MEA		68.201	241.959	9.157	0.983	8.99
5%	(+)-3-Carene	91.613	65.04	5.152	0.978	12.16
	(±)-α-Pinene	81.624	416.282	7.54	0.978	12.00
	(−)-β-Pinene	79.843	419.731	7.663	0.982	11.82
	(+)-Limonene	112.499	111.587	4.605	0.979	15.36
	Turpentine	93.140	124.781	5.762	0.973	12.56
	Mix-monoterpene	91.486	126.549	5.84	0.974	12.43
10%	(+)-3-Carene	89.768	125.2	5.9	0.976	12.28
	(±)-α-Pinene	81.116	488.797	7.672	0.986	12.11
	(−)-β-Pinene	81.752	192.869	6.733	0.983	11.72
	(+)-Limonene	88.877	179.027	6.114	0.999	12.73
	Turpentine	89.513	203.785	6.406	0.972	12.45
	Mix-monoterpene	91.040	86.253	5.46	0.977	12.25
20%	(+)-3-Carene	91.995	87.883	5.45	0.975	12.32
	(±)-α-Pinene	81.688	344.178	7.291	0.986	12.02
	(−)-β-Pinene	82.197	353.544	7.274	0.987	12.10
	(+)-Limonene	80.416	691.62	7.984	0.995	12.29
	Turpentine	93.776	115.599	5.659	0.972	12.59
	Mix-monoterpene	86.014	322.104	7.035	0.973	12.31

Logistic equation: *Y = K/(1 + ae^−bt^)*, *Y* means size of the colony (cm^2^), t means culture time, **K** is maximum size of the colony (cm^2^), **a** is parameter and **b** is maximum of relative growth rate (cm^2^/day).

**Table 6:** Statistics significant of RGSs expression from *L. qinlingensis* in different terpenoids.

Gene	(+)-3-Carene		(±)-α-Pinene		(−)-β-Pinene		(+)-Limonene		Turpentine		Mix-Monoterpene	
	F	Sig	F	Sig	F	Sig	F	Sig	F	Sig	F	Sig
*LqFlbA*	5.817	0.061	0.455	0.728	9	**0.029**	20.224	**0.007**	4.257	0.098	4.452	0.092
*LqRax1*	1.753	0.294	7.841	**0.038**	2569	0.192	2.642	0.186	43.679	**0.002**	8.591	**0.032**
*LqRgsA*	5.045	**0.045**	18.965	**0.008**	9.841	**0.026**	29.271	**0.004**	23.106	**0.005**	17.967	**0.009**

Values in bold indicate significant difference among different concentrations of the same stimulus with one-way ANOVA (α = 0.05). Multiple comparisons among different terpenoids with Tukey tests are shown in Figure 4 with different letters.

## Data Availability

Data is contained within this article.

## Supplementary Materials

The following supporting information can be downloaded at: https://www.mdpi.com/article/10.3390/microorganisms10091698/s1, Figure S1. The main steps of terpenoid treatment. Figure S2. Domain structure analysis of three putative RGS proteins in *L. qinlingensis*.
