# Supplementary material for: Phylogeny of Regulators of G-Protein Signaling Genes in Leptographium qinlingensis and Expression Levels of Three RGSs in Response to Different Terpenoids
Source: Microorganisms. 2022 Aug 24;10(9):1698. doi: 10.3390/microorganisms10091698 (PMC9506272; doi:10.3390/microorganisms10091698)
Supplement: Supplementary file 1 [file microorganisms-10-01698-s001.zip › microorganisms-1849877-supplementary.pdf]

microorganisms

Supplementary Materials

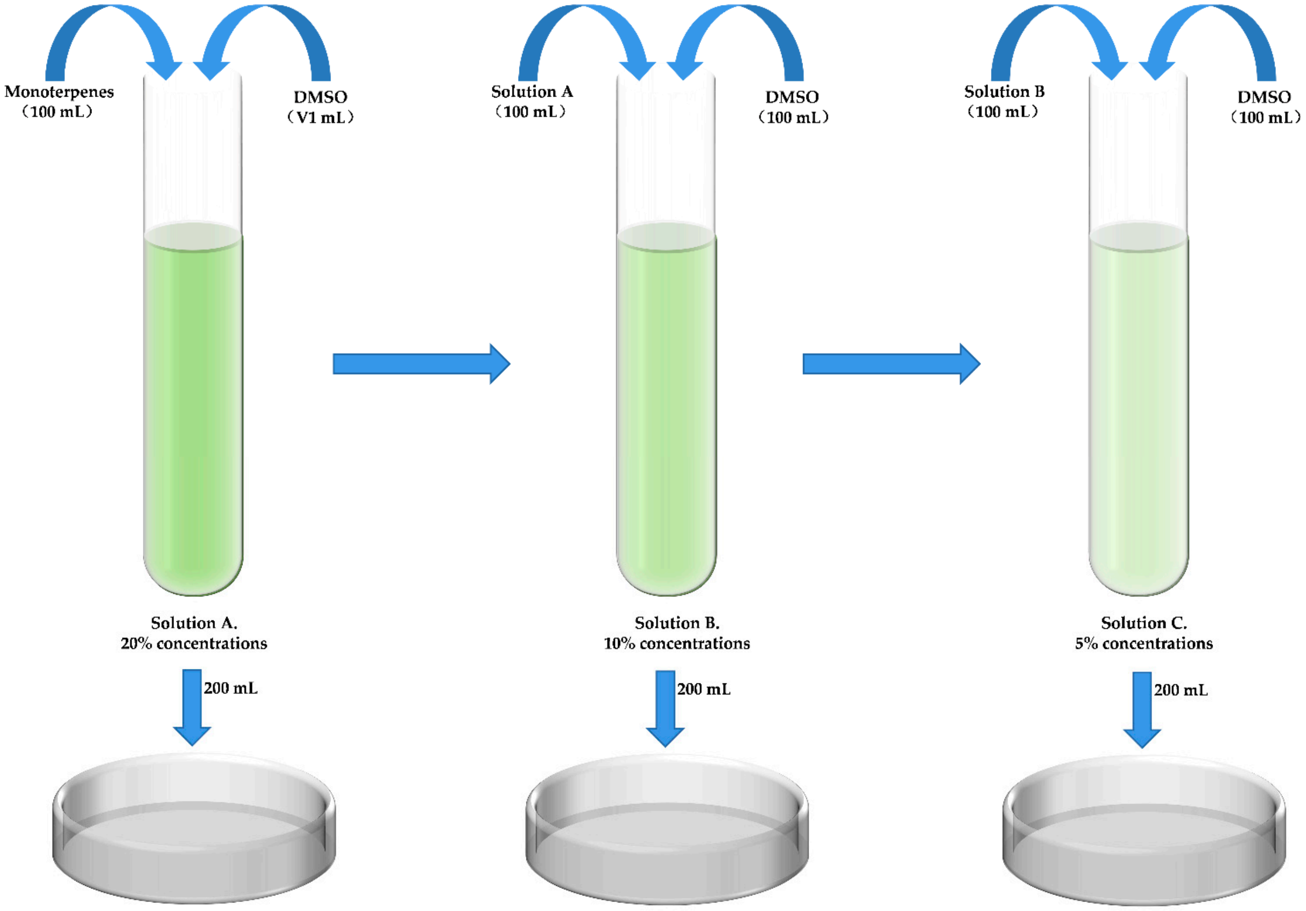

Figure S1. The main steps of terpenoid treatment. Add 100 mL of terpenoid stock solution and V1 (Table 1) volume of DMSO to the burette, and after mixing, it is a 20% concentration of terpenoid dilution (solution A). Take 100 mL of solution A into a new burette, add 100 mL of DMSO, and mix to obtain a 10% concentration of terpenoid dilution (solution B). Take 100 mL of solution B into a new burette, add 100 mL of DMSO, and mix to obtain a 5% terpenoid dilution (solution C). Pipetting 200 mL of each concentration test chemical onto the centre of each MEA medium overlaid with cellophane and gently swirling over the agar surface before inoculation.

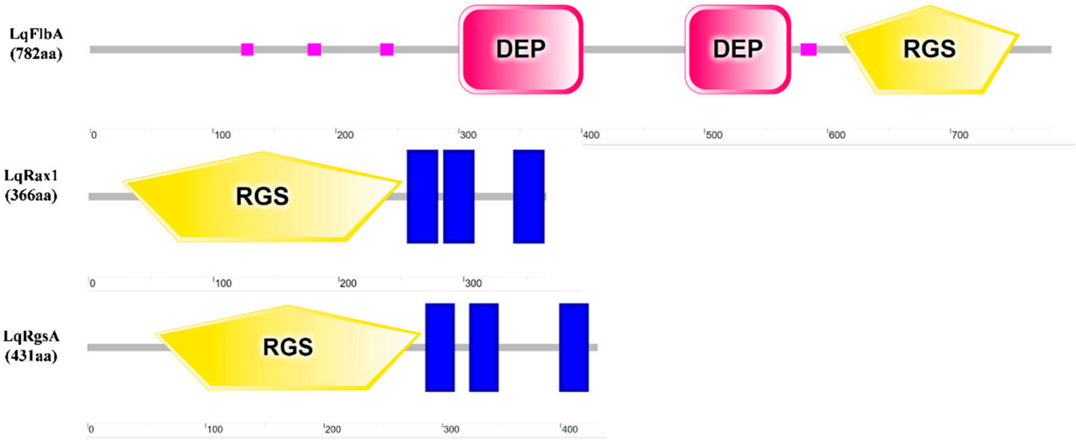

Figure S2. Domain structure analysis of three putative RGS proteins in *L. qinlingensis*. They were identified using the SMART online tool and constructed using DOG version 2.0 software.
